# 6.D. Workshop: Strengthening awareness of obesity in Europe: from knowledge to action

**DOI:** 10.1093/eurpub/ckac129.353

**Published:** 2022-10-25

**Authors:** Organised by: EUPHA-FN, EUPHA-CHR, WHO Europe, Chair persons: Iveta Nagyova (EUPHA), Licia Iacoviello (Italy)

## Abstract

The obesity rates in Europe are constantly growing with a substantial input from the market towards unhealthy food products, a general carelessness or lack of knowledge of the general population, and a major drive of modern societies towards an obesogenic environment. With about half of European people having weight problems, strengthening the resources across institutions, scientific associations, and governmental bodies is highly warranted to try to counteract this major public health issue, which seems to have played a pejorative role over the last 2 years regarding the health risks related to the COVID-19 pandemic. Emerging evidence provides a more complex and multifaced understanding of obesity that it should be considered a disease far beyond the simple concept of excess of body weight, while characterized by a constellation of related comorbidities connecting chronic conditions. Excess body weight is considered a risk factor for cardio-metabolic diseases and certain cancers, but investigating the issue with a more holistic approach would add to this context also the psychosocial burden and a broader involvement of the immune system and inflammatory processes potentially responsible for most obesity-related chronic diseases. Scientific research is also investigating the role of diet-related nutritional and non-nutritional risk factors that may play a role in obesity and its health consequences, also in the context of European countries and the resulting differences depending on the traditional dietary patterns adopted. Moreover, current studies are exploring how dietary choices impact the environment aiming to a reform and a rethinking of the food systems, of citizen/customer preferences, and ultimately of dietary habits to invert the controversial long-term projections that put at risk the global environmental sustainability. The EUPHA Food and Nutrition Section, the Chronic Diseases Section, in collaboration with the World Health Organization (WHO), aim to propose a joint workshop to promote the current knowledge from the latest research on obesity and related chronic conditions, including an objective and more complex biological and societal examination of this public health problem, with a focus on diet-related risk factors and an analysis of how dietary choices may impact the environment. The workshop will have its focus on the presentation of the new WHO European Regional Obesity Report accompanied with ground-breaking new research in the field. The experts will aim to provide new perspectives on a well-known public health issue, discussing about current strategies to tackle the obesity pandemic and possible scenarios for the near future.

**Key messages:**

• Obesity should be considered as a disease, not limited to excess body weight but investigated in a more complex context of comorbidities.

• Current research should also approach the study of obesity with a more holistic approach, including dietary factors and environmental impacts.

